# Effect of Microwave Roasting and Extraction Solvents on the Bioactive Properties of Coffee Beans

**DOI:** 10.1155/2021/4908033

**Published:** 2021-09-21

**Authors:** Ahmad Mohammad Salamatullah, Khizar Hayat, Fohad Mabood Husain, Mohammed Asif Ahmed, Shaista Arzoo, Abdullah Mohammed Alghunaymi, Abdulhakeem Alzahrani, Heba Kahlil Alyahya, Nawal Al-Badr, Mohammed Bourhia

**Affiliations:** ^1^Department of Food Science & Nutrition, College of Food and Agricultural Sciences, King Saud University, P. O. Box 2460, Riyadh 11451, Saudi Arabia; ^2^Laboratory of Chemistry‐Biochemistry, Environment, Nutrition and Health, Faculty of Medicine and Pharmacy, Hassan II University, Casablanca 5696, Morocco

## Abstract

Coffee is an intricate mixture of thousands of chemical compounds that are accountable for its flavor and aroma. Roasting is a key step in the processing of coffee beans. This study assessed the effect of microwave roasting (MW) and extraction solvents (ES) on the total polyphenol content, total flavonoid content, and antioxidant activity of coffee beans. The untreated and microwave-roasted (MR) coffee beans showed a total polyphenol content of 40.40 and 35.15 mg GAE/gm DW, respectively, when methanol was used as the solvent for extraction. Similarly, for the untreated coffee beans, the methanol extracted coffee had a significantly (*p* < 0.05) higher total flavonoid content (39.34 mg CE/g DW) as compared to ethanol (34.82 mg CE/g DW). The obtained IC_50_ for the untreated and microwave-roasted samples as extracted by methanol were 4.13 and 5.68 mg/mL, respectively, while the IC_50_ values of untreated and microwave-roasted samples extracted by ethanol were 4.59 and 6.24 mg/mL, respectively. Untreated coffee beans exhibited a higher reducing power (1.237) than that of the microwave-roasted ones (0.839) when extracted with methanol. Chlorogenic acid was the major (2.31–2.68%) phenolic compound found in all the coffee samples whether it was untreated or microwave-roasted. Vanillin demonstrated the lowest (0.118–0.166%) phenolic compound found in the coffee bean samples. These results might be helpful for obtaining the maximum health benefits from coffee.

## 1. Introduction

Plant-derived foods have been used since the dawn of humankind for promoting health and preventing diseases. People from different cultures worldwide have revered coffee not only for its aromatic compounds but also because of its stimulating and health-promoting properties [1]. In addition to being the world's most highly consumed beverage, coffee has high nutritional value [2]. Many countries grow coffee as a primary crop as well as a valuable commodity [3–5]. Even though studies have reported inconsistent results in connection with coffee consumption, the general consensus is that regular, moderate coffee consumption by healthy individuals is either benign or slightly beneficial [6–10]. Coffee health benefits include reduction in the risk of metabolic syndrome, and protection against noncommunicable diseases such as liver disease, diabetes, cancer, and Parkinson's disease [11–13]. Polyphenols are abundant micronutrients in our diets, and evidence for their role in the prevention of noncommunicable diseases is growing. The bioavailability of polyphenols in coffee differs from one type to another [14]. High amounts of antioxidants and bioactive compounds are found in coffee, and it is the major source of chlorogenic acid [15]. Intake of chlorogenic acid varies widely but may be very high, up to 800 mg/d among coffee drinkers [16]. A single cup of Arabica coffee contains around 70 to 200 mg of chlorogenic acid [17]. Coffee contains a large number of bioactive compounds which are presented in Table 1 [18–20].

Raw coffee beans undergo a chemical transformation during roasting and various factors can influence the biochemical composition of the end product. These factors include but are not limited to the type of beans, method of preparation and degree of roasting [21,22]. The coffee roasting process creates a special aroma and taste [23]. In addition, aroma and flavor are affected by temperature and time and the roasting parameters of coffee [18]. Longer roasting of coffee influences the level of bioactive compounds [24]. In a study by Alkaltham, the total phenol content of green coffee beans was reduced by 13.59% and 16.66% on microwave and oven roasting respectively [25]. There is an inverse relationship between the degree of roasting and antioxidant content [26]. Thus, the coffee roasting process is a key factor for maintaining nutritional value [18]. The phytochemicals in the food matrix have the property of being soluble in specific solvents, although, there is no universally accepted procedure available for measuring the antioxidant and phenolic contents of food. This in turn increases the need for careful selection of the extracting solvent [27,28]. Coffee extraction is a process, which states to dissolving the soluble components of coffee beans powder in a liquid solvent. In a study on comparing the results from water and methanolic extracts of coffee from different countries, the highest amount of phenolics, caffeine, reducing power, ability to chelate Fe2+, inhibition of linoleic acid peroxidation, and inhibition of lipoxygenase was determined for a methanolic extract of coffee [29]. An efficient method of preserving the bioactive compounds is needed [30]. Solid-liquid extraction with different solvents or solid-phase extraction (SPE) followed by high-performance liquid chromatography (HPLC) for the determination of phenolic compounds is reported [31]. HPLC is commonly used for the qualitative and quantitative determination of phenolic compounds in coffee beans and HPLC based methods use mainly C18 with 5 *μ*m particle size packing materials as the stationary phase [32]. A lot of studies have been conducted on coffee; thus, data on the effects of microwave roasting with different extraction solvents on the bioactivity of coffee grown in Saudi Arabia is hardly available. As a result, this study focused on analyzing the effect of microwave roasting (MW) and extracting solvents (ES) on the bioactivity of coffee beans. Therefore, the total polyphenol content (TPC), total flavonoid content (TFC), and antioxidant activity (AA) in terms of DPPH (2, 2‐diphenyl‐1‐picryl‐hydrazyl), reducing power, and identification of phenolic compounds will be detected.

## 2. Materials and Methods

### 2.1. Materials

*Coffea arabica* was obtained from the Jazan region located in the Kingdom of Saudi Arabia. The coffee beans were sun-dried until their moisture level reached 11.2% of the dry weight. The samples were then ground and passed through a 60-mesh (250 *μ*m) sieve. Coffee bean powder (4 g) was heated using a microwave oven at 720 W for 6 min. An unheated sample was used as a control.

### 2.2. Extraction of the Samples

**Figure 1:**
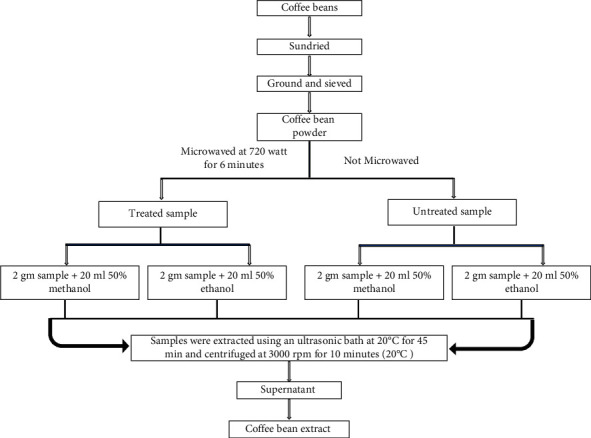
Schematic representation of preparation of coffee bean extract.

Two grams of coffee bean powder was extracted with 20 mL of either 50% methanol or 50% ethanol using an ultrasonic bath at (20°C) for 45 min. Afterward, the sample mixture was centrifuged for 10 minutes at 3000 rpm and the temperature of the machine was set at 20°C. Finally, the obtained supernatant was filtered through filter paper (Whatman filter paper No. 2). The coffee bean extract (CBE) was maintained at 4°C and used for antioxidant assays. Steps followed in preparation of coffee bean extract are presented in Figure 1.

### 2.3. Total Polyphenol Content

The method suggested by Hayat [33] was used to analyze TPC. In brief, 25 *μ*L of CBE was mixed with 1500 *μ*L of water. After this, 125 *μ*L undiluted Folin-Ciocalteu reagent was added to the mixture. After 1 min, 375 *μ*L of 20% sodium carbonate and 475 *μ*L of water were added and the mixture was incubated for 30 min at 23°C. Finally, the absorbance of the mixtures was read at 760 nm and the result was represented as mg gallic acid equivalent per gram dry matter (mg GAE/g DW).

### 2.4. Total Flavonoid Content

The procedure used by Hayat [33] was followed for analyzing TPC in CBE. Two hundred and fifty *μ*L of CBE was mixed with 1000 *μ*L of water, and then 75 *μ*L of both NaNO2 and AlCl3 was added. The mixture was held for 5 min at 23°C, and then 500 *μ*L of 1 M NaOH and 600 *μ*L of water were added. The absorbance of the solution was recorded at 510 nm. The result was represented as mg catechin equivalents per gram dried extract (mg CE/g DW).

### 2.5. DPPH Scavenging

The free radical scavenging capacity of the CBE was determined with 2, 2-diphenyl-1-picrylhydrazyl (DPPH) solution as described by Noreen et al.[34] with slight modification. Briefly, an aliquot of extract (130 *μ*L) and 0.1 mM DPPH solution was mixed thoroughly and allowed to stand in a dark place for 30 min. Afterward, the absorbance of the sample and control was recorded at 510 nm. The control was prepared in the same manner except that methanol was used instead of the coffee extract. Methanol was used as a blank. The DPPH scavenging percentage was measured as follows:

(1) $$\begin{array}{c} \text{DPPH scavenging }\%=\frac{A_{\text{control}}-A_{\text{sample}}}{A_{\text{control}}}\times 100. \end{array}$$

The outcome was illustrated as 50% inhibitory concentration (IC_50_) of the CBE.

### 2.6. Reducing Power

The ferric reducing power of CBE was estimated according to the method of Hayat et al. [35]. Coffee bean extract (0.5 mL) was mixed thoroughly with 1.25 mL buffer (0.2 M, pH 6.6) and 1.25 mL of potassium ferricyanide and incubated for 20 min at 50°C. Then, 1.25 mL of trichloroacetic acid (TCA) was added. Afterward, the CBE mixture was centrifuged for 10 min (at room temperature) at 3000 ×g. An aliquot (1.25 mL) was taken from the supernatant, to which 1.25 mL water and 0.25 mL of ferric chloride were added, respectively. In the end, the absorbance of the sample was measured at 700 nm.

### 2.7. HPLC Analysis of Phenolic Compounds

**Figure 2:**
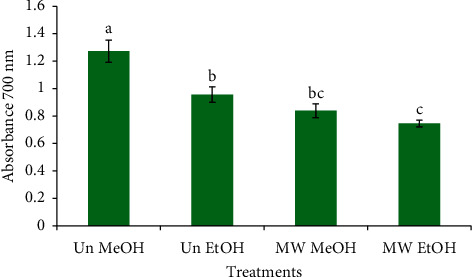
Impact of microwave roasting and extraction solvents on the reducing power of coffee beans. Bars with different small letters are significantly different from one another (*p* < 0.05). Un MeOH: untreated sample extracted with 50% (v/v) methanol, Un EtOH: untreated sample extracted with 50% (v/v) ethanol, MW MeOH: microwave-roasted sample extracted with 50% (v/v) methanol, and MW EtOH: microwave-roasted sample extracted with 50% (v/v) ethanol.

In the current study, utilizing HPLC with the method described previously [36], the phenolic (chlorogenic acid, gallic acid, vanillin, salicylic acid, and caffeic acid) compounds in CBE were quantified. In HPLC system Shimadzu, prominence (Kyoto, Japan) equipped with an LC-20AB binary pump, variable Shimadzu SPD-10A UV-Vis detector was used. The column used was Zorbax SB-C18 (250 × 4.6 mm, 5 *μ*m) (Agilent, Santa Clara, CA, USA) and the mobile was (0.1% formic acid, A) and MeOH (0.1% formic acid, B). The gradient program was the following: 0 min 5% B; 4 min 5%B; 20 min 73% B; 50 min 95% B; 57 min 1% B; 58 min 1% B; 60 min 5% B; at a low rate of 0.7 mL/min. The injection volume was 10 *μ*L, and the detector was set at 280 nm. Compounds were identified by comparing their retention time with those of the standard (Figure 2). All samples were analyzed in duplicate.

### 2.8. Statistical Analysis

Each test was performed in triplicate. The results are illustrated as the mean ± SD (standard deviation). Using SAS statistical software (version 9.2, 2000–2008; SAS Institute Inc., Cary, NC, USA), one-way analysis of variance (ANOVA) was applied to groups, and Duncan's multiple range tests were used to calculate significant differences among the parameters.

## 3. Results and Discussion

### 3.1. Effect of Microwave Roasting (MW) and Extracting Solvents (ES) on the Total Polyphenols of Coffee Bean Extracts

**Figure 3:**
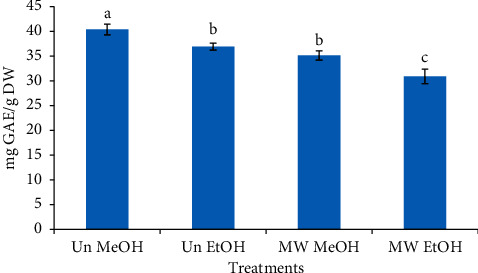
Impact of microwave roasting and extraction solvents on the total polyphenol content of coffee beans. Bars with different small letters are significantly different from one another (*p* < 0.05). Un MeOH: untreated sample extracted with 50% (v/v) methanol, Un EtOH: untreated sample extracted with 50% (v/v) ethanol, MW MeOH: microwave-roasted sample extracted with 50% (v/v) methanol, and MW EtOH: microwave-roasted sample extracted with 50% (v/v) ethanol.

Figure 3 depicts the effect of MW and ES (ethanol and methanol) on the TPC of untreated (raw) and microwave-roasted coffee beans. In the current study, it was found that the untreated CBE exhibited higher TPC as compared with that of microwave-roasted coffee beans as extracted with both methanol and ethanol. It was found that in the coffee beans extracted with methanol, the TPC of the untreated sample was 40.40 mg GAE/g DW, which was higher than the TPC found in the MW sample (35.15 mg GAE/g DW). Previous studies on the impact of roasting on TPC and antioxidant activity have reported inconsistent findings. In accordance with the current report, another study demonstrated that green coffees (Arabica and Robusta) had higher TPC and possessed enhanced antioxidant activity compounds in comparison to roasted coffees [37]. These results are also in accordance with the findings of Nebesny and Budryn who found that green coffee exhibited a higher antioxidant activity as compared to the conventional and microwave-roasted samples [38]. The degradation, polymerization, and auto-oxidation of the phenolic compounds during roasting process might be the cause of a decrease in their content [39]. In another study, in contradiction of the above mentioned studies, statistically insignificant differences were reported between the TPC of raw and roasted coffee beans [40]. However, during the process of roasting, Divišet al.[41] reported an upsurge in the TPC of green coffee beans, and Król et al.[19] reported that the highest TPC was determined in coffees roasted under light and medium roasting conditions.

When the extracting solvent was taken into account, it was found that compared to ethanol, the other solvent (methanol) extracted a significantly higher content (*p* < 0.05) of polyphenols from both the untreated and MW coffee beans. The TPC of untreated coffee beans extracted by methanol and ethanol was measured as 40.40 and 36.92 mg GAE/g DW, respectively. The outcomes of this study are in accordance with the findings of Jaiswal et al.[42] who reported the highest TPC in methanolic extract compared to others, that is, water, acetone, and ethanol extracts.

### 3.2. Effect of Microwave Roasting (MW) and Extraction Solvents (ES) on the Total Flavonoid Content of Coffee Beans

**Figure 4:**
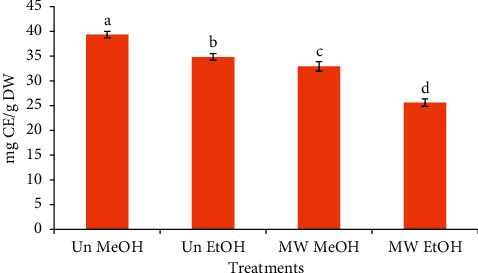
Impact of microwave roasting and extraction solvents on the total flavonoid content of coffee beans. Bars with different small letters are significantly different from one another (*p* < 0.05). Un MeOH: untreated sample extracted with 50% (v/v) methanol, Un EtOH: untreated sample extracted with 50% (v/v) ethanol, MW MeOH: microwave-roasted sample extracted with 50% (v/v) methanol, and MW EtOH: microwave-roasted sample extracted with 50% (v/v) ethanol.

The TFC of CBE is shown in Figure 4. The MW, as well as ES, showed a similar trend for the TFC of coffee beans as was that for the TPC. Untreated coffee beans and methanol as an ES showed a higher TFC as compared to MW coffee beans and ethanol, respectively. The TFCs of untreated and microwave-roasted coffee beans extracted with ethanol were found as 34.82 and 25.59 mg CE/g DW, respectively. When comparing the results of this study to the findings of Ghafoor et al.[43], it was found that roasting has similar effects on other plant materials as he reported a decrease in the TFC of poppy seeds and oil upon roasting in the microwave oven at 720 W for 5 min. In contrast, Al-Juhaimi et al. [44] indicated that the TFC of apricot kernels was increased by roasting them at 320 W, 540 W, but decreased when the microwave power was increased to 720 W. Microwave heating breaks open the cell walls of the plant materials, allowing phytochemicals to be released more easily, and increasing the availability of bioactive materials [45].

Methanol extracted resulted in a significantly (*p* < 0.05) higher total flavonoid content when compared to ethanol. For instance, the TFC of untreated coffee beans as extracted by methanol and ethanol was 39.34 and 34.82 mg CE/g DW, respectively. In an earlier study, extraction was done with acetone, water, ethanol, and methanol and highest level of TFC was obtained using methanol as an ES, which corroborates well with the findings of the present study [42]. Polarity is responsible for the extraction of phenolic compounds from solvents. Alkalthamet al. reported higher TPC and TFC in coffee beans and pulp samples extracted with methanol as compared to ethyl acetate [46]. Similarly, in another study conducted on green coffee beans extracted in ethanol and ethyl acetate, the ethyl acetate extract exhibited a lower TPC [47].

### 3.3. Effect of Microwave Roasting (MW) and Extraction Solvents (ES) on the DPPH Scavenging of Coffee Beans

**Figure 5:**
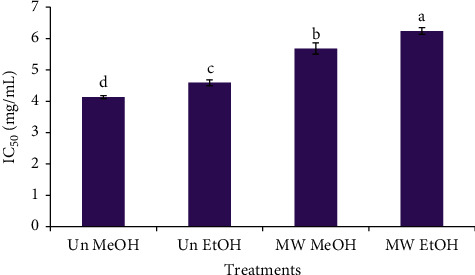
Impact of microwave roasting and extraction solvents on the DPPH scavenging of coffee beans. Bars with different small letters are significantly different from one another (*p* < 0.05). Un MeOH: untreated sample extracted with 50% (v/v) methanol, Un EtOH: untreated sample extracted with 50% (v/v) ethanol, MW MeOH: microwave-roasted sample extracted with 50% (v/v) methanol, and MW EtOH: microwave-roasted sample extracted with 50% (v/v) ethanol.

The antioxidant activity of the untreated and MW coffee beans was measured using the 2,2-diphenyl-1- picrylhydrazyl (DPPH) radical scavenging method and represented as 50% inhibitory concentration (IC_50_) of the coffee extract. The results expressed in Figure 5 showed that MW and the ES had a significant (*p* < 0.05) effect on the DPPH scavenging of the coffee beans. In comparison to the MW coffee beans, the untreated samples of CBE exhibited a significantly higher antioxidant activity with lower IC_50_ values. For example, the obtained IC_50_ values for untreated and MW samples as extracted by methanol were 4.13 and 5.68 mg/mL, respectively. Similarly, Doğan et al.[48] found that the roasting of coffee exerted a negative effect on its antioxidant potential. In another study, green (raw) coffee also resulted in a higher antioxidant activity when compared to the conventional and microwave-roasted samples [38]. In contrast, Ludwig et al., [49] stated an upsurge in the DPPH scavenging of the roasted coffee. The DPPH scavenging of some other plant materials like citrus peels and pomace, fennel seeds [45], and apricot kernels [44] was increased by microwave roasting. High-temperature processing is thought to result in an increase of TPC and TFC in the plant materials, which leads to their increased antioxidant activity [50].

In addition, the methanol extracted coffee bean samples had significantly higher antioxidant activity in comparison with their ethanol extracted counterparts. The IC_50_ values for MR samples extracted using methanol and ethanol were 5.68 mg/mL and 6.24 mg/ml, respectively. The methanol extraction of coffee silver skin yielded a higher DPPH quenching ability than that extracted with other solvents [46].

### 3.4. Effect of Microwave Roasting (MW) and Extraction Solvents (ES) on the Reducing Power of Coffee Beans

The outcome of the effect of MW and ES on the reducing power of coffee beans is described in Figure 2. Untreated coffee beans exhibited higher reducing power (1.237) than that of the microwave-roasted ones (0.839) when extracted with methanol. Extraction solvents also showed a significantly different (*p* < 0.05) effect on the reducing power of coffee beans. It was noted that the reducing power of the untreated coffee beans sample extracted with ethanol (0.956) was less than those extracted with methanol (1.237). The results of antioxidant assays (DPPH scavenging, reducing power) echoed the results of TPC and TFC showing that the antioxidant potential of coffee beans was at least in part due to the polyphenol and flavonoid contents.

A previous study [51] reported a negative trend of the antioxidant capacity of *Coffea arabica* with increasing roasting degrees, which is in accordance with the results of the present study. Likewise, our findings find support from the observations made with green coffee beans decline that demonstrated a decline in the antioxidant capacity during the roasting process [52]. However, in contrast to our results, when compared to green coffee, Liang et al.[53] reported an increase in the antioxidant capacity of roasted beans. Such inconsistencies could be accredited to the intricacy of the chemical reactions during the roasting process. The plausible reason could be that during the roasting process of coffee beans, some of the bioactive compounds like chlorogenic acids are degraded which in turn could reduce the antioxidant activity of the coffee beans [52]. In addition, such degradations may also result in the release of other bioactive compounds such as hydroxycinnamates and quinic acid, which contribute to increased antioxidant potential [54]. Moreover, the Maillard reaction may also take place due to high temperature during the roasting process, generating a number of compounds, which can contribute to the elevated antioxidant potential of the product [55]. Consequently, all of the substances appearing during roasting process can either compensate for the loss of some compounds or even contribute towards the enhancement in the antioxidant potential [49].

### 3.5. HPLC Analysis of Phenolic Compounds

**Figure 6:**
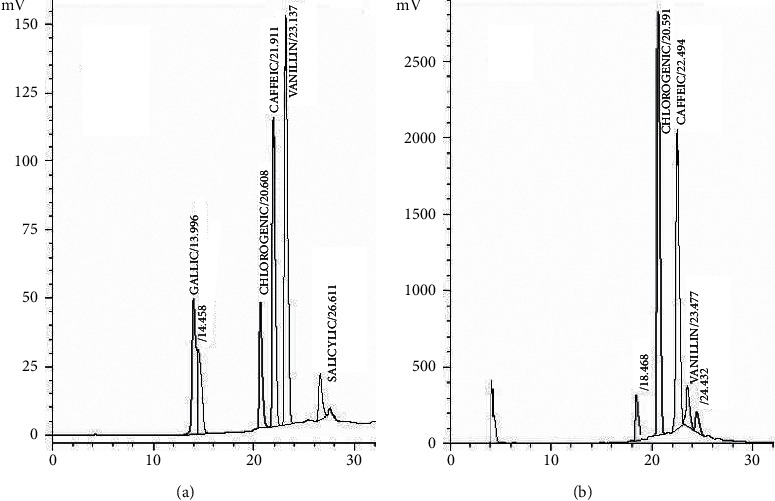
Typical HPLC chromatogram of standards (a) and coffee sample (b).

The average quantitative data reported in this study (Table 2) show that chlorogenic acid was the major (2.31–2.68%) phenolic compound found in all the coffee samples (untreated and microwave-roasted) followed by caffeic acid (0.997–1.18%). Vanillin demonstrated the lowest (0.118–0.187%) phenolic compound found in the coffee bean samples. Typical chromatogram of HPLC for standards and the sample is shown in Figure 6. An insignificant influence of MW and ES on the phenolic compounds of the coffee beans was observed. There is no significant difference between the untreated methanolic (0.997–2.31 g/100 g) or ethanolic (0.997–2.31 g/100 g) extraction and microwave methanolic (0.187–2.68 g/100 g) or microwave ethanolic (0.166–2.55 g/100 g) treatments on the phenolic compounds. Similarly, the individual phenolic compounds, chlorogenic acid, caffeic acid, and vanillin, have close similarity in concentrations of all extraction and treatment methods (Table 2). Traditional heating at a high temperature of 120°C even for a short time results in the loss of 15–36% of the bioactive compounds [56]. Similar results have been reported in Ethiopian and Ugandan with roasted coffee where chlorogenic acid was present in the highest concentrations [44]. In another study, chlorogenic acid was reported as the major phenolic acid in spent coffee grounds extract as confirmed by HPLC [45]. Higher chlorogenic acid levels were extracted by multistep whole coffee fruit extracts than by the single-step extract [46].

## 4. Conclusions

Roasting is an important step in the processing of coffee before it is consumed as a beverage. Therefore, the effects of MW and ES on the TPC, TFC, and antioxidant activity of coffee beans were evaluated. Compared to the untreated coffee beans, the MR coffee beans showed significantly lower TPC, TFC, and antioxidant activity. Similarly, the methanol showed better results compared to ethanol for both untreated and microwave-roasted samples. Irrespective of the ES and MW treatment, all major and important phenolic compounds were present with no significant difference. Chlorogenic acid is the key phenolic acid in untreated, microwave, methanol, and ethanolic extracts and can reap health benefits. This information might be helpful for the processing of coffee beans and can be exploited by the beverage industry. There are certain inconsistencies in the findings as compared to previous studies, but these can be attributed to factors such as method of extraction, microwave treatment, and climatic conditions. Moreover, future studies need to focus on the underlying mechanism of action of roasting on the studied parameters.

## Figures and Tables

**Table 1:** Composition of bioactive compounds of roasted coffee bean [18–20].

Bioactive compound	Traditional roasted
Caffeine	526 ± 1.97
Gallic	117 ± 0.57
Chlorogenic	600 ± 1.83
Quercetin	7 ± 0.03
Kaempferol	6 ± 0.04
Caffeic	5 ± 0.07
Salicylic	12.1 ± 0.16
Epigallocatechin gallate	28 ± 0.19
Quercetin-3-O-glucoside	2.8 ± 0.1
Kaempferol-3-O-glucoside	35 ± 0.15
Caffeoylquinic acid	3530 ± 0.02
Caffeine	2840 ± 0.00
Melanoidins	2380 ± 0.77
Trigonelline	1000 ± 0.00
Redutores	100 ± 0.00
Cafestol	742 ± 41
Nicotinic acid	19 ± 3
Kahweol	465 ± 18

^*∗*^Results are expressed in means ± standard deviation and given in mg/100 g of sample.

**Table 2:** Concentration of phenolics (gm/100 gm) dry weight basis.

Sample	Chlorogenic acid	Caffeic acid	Vanillin
Untreated MeOH	2.31 ± 0.37	0.997 ± 0.33	0.118 ± 0.65
Untreated EtOH	2.31 ± 0.58	0.997 ± 0.67	0.166 ± 0.27
MW MeOH	2.68 ± 0.63	1.18 ± 0.91	0.187 ± 0.63
MW EtOH	2.55 ± 0.87	1.09 ± 0.65	0.166 ± 0.87

Results are expressed as mean ± SD.

## Data Availability

Data used to support the findings are included within the text.
